# Gender, Socioeconomic Status, Race, and Ethnic Disparities in Bystander Cardiopulmonary Resuscitation and Education—A Scoping Review

**DOI:** 10.3390/healthcare12040456

**Published:** 2024-02-10

**Authors:** Audrey L. Blewer, Blair L. Bigham, Samantha Kaplan, Marina Del Rios, Marion Leary

**Affiliations:** 1 Department of Family Medicine and Community Health, Duke University School of Medicine, Durham, NC 27710, USA; 2 Department of Population Health Sciences, Duke University School of Medicine, Durham, NC 27710, USA; 3 Dalla Lana School of Public Health, University of Toronto, Toronto, ON M5R 0A3, Canada; 4 Scarborough Health Network Research Institute, Toronto, ON M1P 2T7, Canada; 5 Medical Center Library and Archives, School of Medicine, Duke University, Durham, NC 27710, USA; 6 Carver College of Medicine, University of Iowa, Iowa City, IA 52242, USA; 7 School of Nursing, University of Pennsylvania, Philadelphia, PA 19104, USA

**Keywords:** bystander cardiopulmonary resuscitation, cardiopulmonary resuscitation education, disparities, race, ethnicity, gender

## Abstract

Background: Social determinants are associated with survival from out-of-hospital sudden cardiac arrest (SCA). Because prompt delivery of bystander CPR (B-CPR) doubles survival and B-CPR rates are low, we sought to assess whether gender, socioeconomic status (SES), race, and ethnicity are associated with lower rates of B-CPR and CPR training. Methods: This scoping review was conducted as part of the continuous evidence evaluation process for the 2020 American Heart Association Guidelines for Cardiopulmonary Resuscitation and Emergency Cardiovascular Care as part of the Resuscitation Education Science section. We searched PubMed and excluded citations that were abstracts only, letters or editorials, and pediatric studies. Results: We reviewed 762 manuscripts and identified 24 as relevant; 4 explored gender disparities; 12 explored SES; 11 explored race and ethnicity; and 3 had overlapping themes, all of which examined B-CPR or CPR training. Females were less likely to receive B-CPR than males in public locations. Observed gender disparities in B-CPR may be associated with individuals fearing accusations of inappropriate touching or injuring female victims. Studies demonstrated that low-SES neighborhoods were associated with lower rates of B-CPR and CPR training. In the US, predominantly Black and Hispanic neighborhoods were associated with lower rates of B-CPR and CPR training. Language barriers were associated with lack of CPR training. Conclusion: Gender, SES, race, and ethnicity impact receiving B-CPR and obtaining CPR training. The impact of this is that these populations are less likely to receive B-CPR, which decreases their odds of surviving SCA. These health disparities must be addressed. Our work can inform future research, education, and public health initiatives to promote equity in B-CPR knowledge and provision. As an immediate next step, organizations that develop and deliver CPR curricula to potential bystanders should engage affected communities to determine how best to improve training and delivery of B-CPR.

## 1. Introduction

The International Liaison Committee on Resuscitation estimates the incidence of out-of-hospital sudden cardiac arrest (SCA) to be between 30 and 97 per 100,000. Survival from SCA is low, ranging from 8 to 10% in many communities [1,2]. Receipt of bystander cardiopulmonary resuscitation (B-CPR), where lay rescuers provide chest compressions and/or rescue breaths to circulate oxygen to vital organs and/or apply a public defibrillator before the arrival of professional emergency services, doubles a victim’s chance of survival from SCA, yet studies have demonstrated that B-CPR rates are low (<40%) [3,4,5]. Additionally, survival from SCA is disproportionately lower in populations who have systematically experienced greater obstacles to health based on known social determinants, including gender, socioeconomic status (SES), race, and ethnicity, and these populations are less likely to receive B-CPR [4,6,7,8]. The National Academy of Medicine and the American Heart Association (AHA) have highlighted increasing B-CPR as a crucial national objective [9,10]. However, B-CPR rates dropped in many countries, such as the UK, US, and Canada, during the pandemic, while the incidence of OHCA rose. Therefore, it is critical to understand the disparities associated with low rates of B-CPR and cardiopulmonary resuscitation (CPR) training.

Many studies have focused on health disparities that relate to B-CPR and outcomes from SCA [11,12,13,14], yet a synthesis of the findings as they directly relate to B-CPR and CPR training is lacking. Given the health disparities literature, consideration of gender, SES, race, and ethnicity and their impact on CPR is warranted. Understanding these findings may inform future educational, research, and policy initiatives for public health professionals, resuscitation scientists, and policy makers.

Under the auspices of the AHA 2020 resuscitation guidelines update, we conducted a scoping review to assess whether gender, SES, race, and ethnicity are associated with lower rates of B-CPR and CPR training to inform whether targeted training for these populations is warranted.

## 2. Materials and Methods

### 2.1. Protocol

This work was conducted as part of the continuing evidence review of the Resuscitation Education Science 2020 American Heart Association Guidelines for Cardiopulmonary Resuscitation and Emergency Cardiovascular Care [15]. The writing group solicited topics and ranked ideas based on perceived priority and importance. During this process, guidelines around disparities in resuscitation education were noted as a high-priority area. To address this, the writing group conducted an evidence update review of the topic, which was guided by the Resuscitation Education Science chair and members of the AHA scientific committee.

### 2.2. Objectives

We asked the question “Do racial, ethnic, socioeconomic, or gender disparities impact resuscitation education and/or contribute to barriers in bystander CPR?” We sought to assess whether gender, SES, race, and ethnicity were associated with lower rates of B-CPR and CPR training ultimately to inform whether targeted training for these populations is warranted.

### 2.3. Relevant Definitions

Gender was defined on an individual level as self-identified or clinician-identified male, female, or non-binary. SES was characterized by self-identified income and education by individual and or neighborhood. We defined racial and ethnic populations as marginalized individuals, groups, and neighborhoods who have historically experienced inequity or prejudice, such as Black, Hispanic, and linguistically isolated communities with limited English proficiency in the US.

### 2.4. Outcomes of Interest

Given our review objective, our primary outcomes of interest were B-CPR and CPR training. Thus, we excluded manuscripts where survival to hospital discharge or other surrogate measures were the primary outcome.

### 2.5. Search Methods and Exclusion Criteria

A trained medical librarian searched PubMed from inception to 10 October 2019 using a combination of subject headings and keywords to represent the concepts of CPR, bystanders, education, and healthcare disparities. We excluded abstracts, letters or editorials, and pediatric studies (ages <18 years of age). Because this was a new question of interest, we did not limit our search by date range.

All results were compiled into EndNote X9.3.3 (Clarivate Analytics) and then imported into Covidence (Melbourne, Australia) for screening. The search was updated on 16 June 2020. Additionally, the search was updated, translated, and run in Embase (Elsevier) from inception to 10 October 2019. Search terms are available in Supplementary Table S1.

### 2.6. Selection Process

As an initial measure, two authors reviewed 398 articles from the initial PubMed query. The abstracts were imported into citation management software (Available at www.covidence.org.) (Covidence) and screened as “relevant” or “not relevant” to the predefined objective. During this process, we excluded any manuscript where the primary outcome of interest was not B-CPR or CPR training. From there, A.L.B. and M.L. discussed abstracts where there were potential differences and came to an agreement. Once the final sample was agreed upon, A.L.B. and M.L. read and compiled 24 articles for this scoping review. Based on prior knowledge of the topic, A.L.B. and M.L. decided to add the Anderson et al. article to the scoping review. We acknowledge potential risk of bias including selection bias and publication bias. Measures taken to address this include consulting with an external librarian.

As a secondary measure to ensure adequate coverage of this topic, the PubMed search was updated and translated into Embase (Elsevier). Two authors reviewed the additional items retrieved. This secondary search did not yield additional manuscripts to add to the scoping review.

### 2.7. Data Compilation

Given our objective, we organized the articles by relevant theme. In addition to gender, SES, race, and ethnicity, we decided to add the themes of language and perceptions. Additionally, we organized the articles by methodologies to assist readers with understanding the scope and depth of the current literature.

## 3. Results

**Figure 1:**
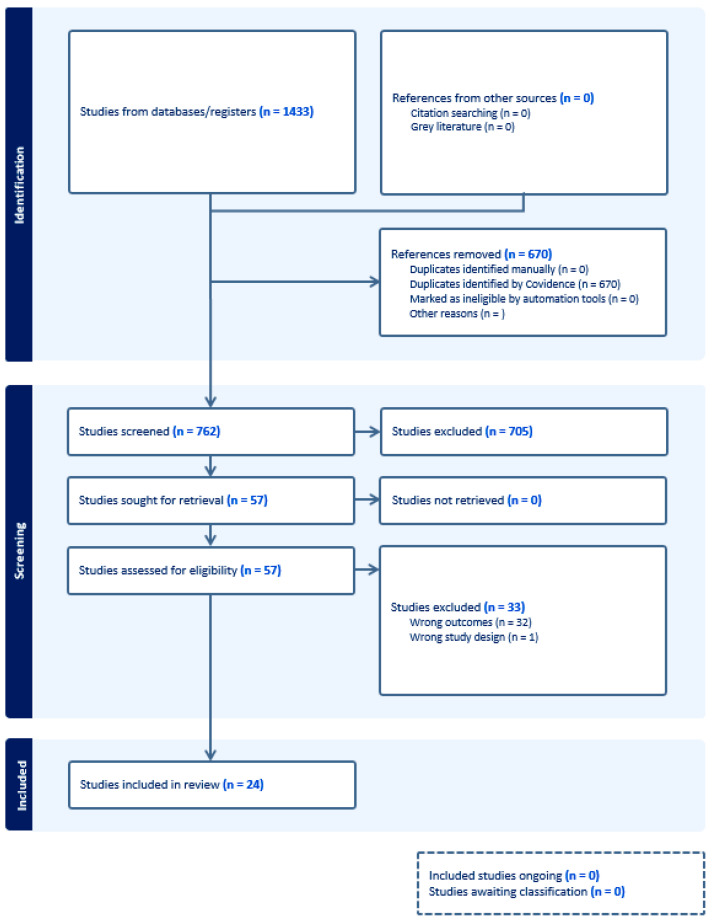
PRISMA Flow Diagram.

We reviewed 762 unique manuscripts; 24 were identified as relevant to our aim (Figure 1). Of the 24 articles, all were primary studies and no previous reviews were identified. The 24 papers were categorized into five themes: gender, SES, race and ethnicity, language, and perceptions. Table 1 summarizes the disparity themes emerging from the literature; 4 studies explored gender disparities in B-CPR and CPR education [16,17,18,19], 12 studies explored SES [20,21,22,23,24,25,26,27,28,29,30,31], and 11 studies explored race and ethnicity, language, and perceptions [25,29,31,32,33,34,35,36,37,38,39]. Three studies spanned two themes [25,29,31].

Table 2 summarizes the methodology by theme. The only randomized control trial was a simulation study exploring gender disparities in B-CPR and CPR education [16]. Thirteen were retrospective cohort studies (five race and ethnicity, language, and perceptions [25,31,32,33,34], nine SES [20,21,22,23,24,25,26,30,31], and one gender [17]); five were survey studies (one race and ethnicity, language, and perceptions [36], two SES [27,28], and two gender [18,19]); three were qualitative studies exploring perspectives on disparities [37,38,39]; one study analyzed association of SES and race with enrollment in B-CPR courses [29]; one study examined the availability of Spanish language B-CPR information on the internet [35].

### 3.1. Gender

Four studies examined the impact of gender on B-CPR (Table 3) [16,17,18,19]. Men are more likely than women to receive CPR in public locations. Studies on this theme explored reasons for this, such as fear of causing harm and discomfort by placing hands on a woman’s chest to perform compressions.

### 3.2. Socioeconomic Status

Twelve studies examined the impact of SES on B-CPR and CPR education (Table 4) [20,21,22,23,24,25,26,27,28,29,30,31]. It was commonly found that B-CPR rates were lower in census tracts or neighborhoods where SES, and particularly the median income and education level were lower. Additionally, survey studies found that B-CPR training rates were lower in these communities. There was a paucity of qualitative work to try to explain disparities.

### 3.3. Race and Ethnicity

Six studies examined the impact of race and ethnicity on B-CPR and CPR education (Table 5) [25,29,31,32,33,34]. One study found a higher rate of B-CPR in white SCA victims [25]. One study found lower rates of B-CPR in Black victims, and one study found lower rates of B-CPR in Hispanic victims [33,34]. Three studies explored neighborhood composition and rates of B-CPR and CPR education [25,31,32]. Specifically, one study found lower rates of B-CPR in neighborhoods with a higher Hispanic composition [32]. Two studies found Black neighborhoods were associated with lower rates of B-CPR [25,31]. One study found lower rates of CPR training in both Black and Hispanic neighborhoods [29]. Some studies linked lower SES to the Black or Hispanic race [25,31].

### 3.4. Language

Two studies reported language disparities in B-CPR training (Table 6) [35,36]. One study found that 16% of CPR training websites offered Spanish content [35]. One study of a Cambodian community found that English proficiency was a predictor of both having taken a CPR class and willingness to activate 9-1-1 [36].

### 3.5. Perceptions

Three focus group studies of marginalized populations reported on barriers to B-CPR (Table 6) [37,38,39]. Cost, fear of legal consequences, distrust of law enforcement, language concerns, fear of liability, and lack of confidence in performing CPR were all reported as barriers.

## 4. Discussion

Disparities in health and healthcare related to gender, SES, race, ethnicity, and language have been documented throughout the medical literature. Numerous studies over decades have shown this to be true for SCA incidence, as well as disparities in implementation of B-CPR by lay responders [7,11,20,40]. As demonstrated in the data, individuals from marginalized racial and ethnic groups and those with low SES have lower rates of both CPR training, as well as receipt of B-CPR. Additionally, victims who were female are less likely to receive B-CPR in public locations than those who were male; lower rates of CPR training also exist in populations with limited English proficiency.

Of those who perform B-CPR, 90% are trained, highlighting the importance of training communities in this lifesaving skill. Among untrained bystanders, there are well-documented reasons B-CPR is not performed more commonly. Insufficient knowledge about the benefit and technique of performing CPR is a significant barrier, as is fear of performing CPR incorrectly and causing harm. Another barrier is fear of contracting communicable disease while performing rescue breaths, which requires the rescuer to put their mouth onto the mouth of the person being rescued.

Potential reasons for the disparities in B-CPR specific to affected populations have been highlighted in multiple low-level studies; in our scoping review of the disparity literature related to B-CPR, we found that the vast majority of studies (53%) were retrospective with no RCTs with actual patients (only one RCT was documented in a simulation setting). There is a growing realization in the resuscitation science field that additional studies need to be implemented if we are to truly examine the cause of these disparities for women and those in Black and Brown communities, as well as to determine the best way to increase equitable SCA care. In order to close these disparity gaps, studies will need to address the complex reasons for this multifaceted problem including the current climate in the United States around sexual assault, cultural norms, and gender misconceptions, as well as the current climate of racial injustice in this country.

The format and nature of CPR training have changed little in the past 40 years; we ask, is it time for a disruption, supported by technology and novel educational methods? To do this, we may consider reimagining the current system of CPR training and work with each of the communities using a human-centered approach, co-collaborating with communities to create and design CPR training programs that work for them. Developers of CPR training programs may consider engaging individual communities to co-design content and develop strategies for delivery. Additionally, we need a diverse and inclusive representation of those who experience SCA and lay responders including women and people of color. We also need to increase the availability of trainings for communities of color and of low-income groups and remove language barriers by increasing the availability of training programs in languages other than English. CPR training programs should be sensitive to the impact that prejudice, systemic racism, and xenophobia have on communities of color, and how these have fed into distrust and fear of emergency response and health systems. These fears must be addressed and trust built between emergency services and communities. All of the factors associated with low B-CPR need to be thoroughly assessed through multifaceted approaches, including community-based participatory research, and then appropriately addressed if we are to decrease disparities in bystander response and have more equitable SCA outcomes.

### 4.1. Gender

One contributing factor for the disparities in B-CPR for female victims could be the “masculinity” of CPR trainings. Standard CPR training videos generally portray the victims of an SCA as male-gendered. One reason for this may have to do in part to cultural sensitivities where men may not be seen touching a woman in public. Media may also play a role as SCA victims on television shows and in movies are often portrayed as male-gendered. In addition, the manikin’s used for CPR are generally manufactured to resemble a male-gendered victim, with a flat chest and short hair.

The underrepresentation of women in training materials and in the media may suggest to the public that women do not suffer an SCA similarly to men, and therefore may not require B-CPR when a female victim collapses and is unresponsive in the out-of-hospital setting. On a societal level, the cultural norm toward objectifying women’s bodies may lead to hesitation on the part of lay responders to provide CPR for fear of being accused of inappropriate touching or sexual misconduct [19]. Public campaigns to address this stigma are needed.

One innovative solution put on the market by an independent company is the Womanikin, a universal attachment of female breasts that can be placed on a standard CPR manikin to represent a female-gendered victim. Another way to remove misconceptions about women’s risk of SCA and normalize B-CPR on female bodies is to increase the representation of women in training materials both as recipients of B-CPR and as first responders. These strategies will be most effective in concert with larger societal movements to promote gender equity and decrease the sexualization of women’s bodies.

### 4.2. Socioeconomic Status

Higher SES is often associated with higher education attainment and therefore a greater opportunity to be exposed to CPR training. Mandates to implement CPR training in schools have been proposed as a strategy to increase B-CPR training in the general population. However, there is a disparate availability of resources in public schools both in rural and urban communities. In the US, local funding for schools largely comes from property taxes. Segregation by income results in a wide variation in property values from district to district, and with that wide disparity in tax revenues comes disparities in property tax revenue used to fund schools. These systemic issues affect access to many educational opportunities, including exposure to CPR training.

CPR training can be cost-prohibitive and difficult to geographically attend, which leads to real financial barriers and time barriers for people in low-SES communities and limited opportunity to learn CPR. Free CPR training and online videos have been developed to train people in B-CPR; however, more research is needed to understand the barriers to those as well, including access to reliable, high-speed internet for online videos and trainings, as well as access to in-person programs [27,41]. We need to democratize CPR training in a way that increases CPR training rates in all communities for all populations. Diversifying the instructor pool to reflect community demographics may also help.

### 4.3. Race and Language

Racial, ethnic, and language proficiency disparities in B-CPR occur in the context of broader historic and contemporary social inequality and are evidence of persistent discrimination against people of color [42]. One of the most significant consequences of systemic racism is fear of emergency services in communities of color [37,43]. We need to be sensitive to marginalized populations who may have a fear of providing bystander CPR or engaging with the 9-1-1 emergency system due to a history of negative interactions with law enforcement. It is our responsibility to learn from and design with marginalized communities to create strategies to better addresses these fears. A multifaceted approach including human-centered community-based participatory research may inform not only strategies for CPR trainings and response during an SCA, but also larger systemic efforts to eliminate maladaptive interactions with emergency response and healthcare systems. Dispatch-assisted CPR should increase linguistic assistance staff to improve access for lay responders with limited English proficiency, and CPR training materials should be made available in multiple languages.

### 4.4. Limitations

Our scoping review was limited to English articles. As is common in scoping reviews, we did not assess risk of bias and were unable to conduct a meta-analysis or make specific data-guided recommendations. There is a risk of publication bias.

## 5. Conclusions

We found 24 studies investigating disparities in B-CPR and CPR training. Gender, SES, race, and ethnicity negatively impact receiving B-CPR and obtaining CPR training, often because of language comprehension gaps between curricula and learners or negative experiences with emergency services. The impact of this is that these populations are less likely to receive B-CPR, which decreases their odds of surviving SCA. These health disparities must be addressed by policy makers, organizations that answer and respond to emergency calls, and organizations that provide CPR training through the lenses of anti-oppression, anti-racism, and diversity and inclusion. Our work can inform future research, education, and public health initiatives to promote equity in B-CPR knowledge and provision. As an immediate next step, organizations that develop and deliver CPR curricula to potential bystanders should engage affected communities to determine how best to improve training and delivery of B-CPR to save more lives.

## Figures and Tables

**Table 1:** Disparity themes emerging from the literature.

Theme	Number of Articles
Gender	4 ([16,17,18,19])
SES	12 ([20,21,22,23,24,25,26,27,28,29,30,31])
Race and Ethnicity	6 ([25,29,31,32,33,34], )
Language	2 ([35,36])
Perceptions	3 ([37,38,39])

SES = socioeconomic status.

**Table 2:** Methodologies by theme.

	Gender	SES	Race and Ethnicity	Language	Perceptions
Randomized controlled trial (simulation)	1 ([16])	0	0	0	0
Retrospective cohort	1 ([17])	9 ([20,21,22,23,24,25,26], [30,31])	5 ([31,32,33,34],)	0	0
Cross-sectional survey	2 ([18,19])	2 ([27,28])	0	1 ([36])	
Qualitative interviews	0	0	0	0	3 ([37,38,39])
Other study	0	1 ([29])	1 ([29])	1 ([35])	

SES = socioeconomic status.

**Table 3:** Gender.

Citation	Method	N	Population	Intervention	Outcome
[16]	RCT	69	Undergraduate students	33 female OHCA simulators Control: 36 male OHCA simulators	Rescuers were more likely to completely bare male simulator’s chest (n = 33/36 91.7%) compared to female simulator (n = 14/33 42.4%). Men (n = 2/15, 13.3%) were significantly less likely to de-robe the manikin than women (n = 12/18 removed or 66.7%).
[18]	Cross-sectional survey	582	18 or older, attending a CPR class		Participants feared causing injury to geriatric, female, and adolescent subjects
Blewer [17]	Retrospective cohort	19,331	18 or older		Males had a 27% increased odds of receiving CPR compare to females in public locations.
[19]	Cross-sectional survey	548	Crowd-sourced, Adults living in the US, able to define CPR correctly		Perceived reasons women receive CPR less often

RCT = randomized controlled trial; OHCA = out-of-hospital cardiac arrest; CPR = cardiopulmonary resuscitation; US = United Statues.

**Table 4:** Socioeconomic Status.

Citation	Method	N	Population	Outcome
[27]	Cross-sectional survey	1703	18 or older, attending a CPR class	Lower SES is associated with a lower likelihood of ever being CPR-trained
[28]	Cross-sectional survey	9022	18 or older, RDD survey	Lower income and education were associated with a lower likelihood of CPR training
[29]	Cross-sectional ecologic study	15,109,467	Age 15 to 80, AHA, Red Cross, and Health & Safety Institute trainings	Counties with lower median household income were associated with lower rates of CPR training
[20]	Retrospective cohort	67,219	People with OHCA, excluded do-not-resuscitate (DNR), those with ROSC before EMS	Greater social deprivation (lower workday population density, higher proportion of people in routine occupations, and lower proportion in managerial role) is associated with a higher incidence of OHCA and low rates of B-CPR
[21]	Retrospective cohort	3573	Adults with non-traumatic OHCA, excluded DNR	OHCA in low SES areas was associated with a lower likelihood of B-CPR and poorer survival
[22]	Retrospective cohort	4009	Presumed cardiac OHCA resuscitated by EMS	OHCA in low-SES areas was associated with a lower likelihood of B-CPR
[31]	Retrospective cohort	1466	OHCA for whom resuscitation was attempted by EMS	Low rates of B-CPR were associated with % living in poverty.
[23]	Retrospective cohort	4482	Adults with cardiac causes; excluded EMS-witnessed and arrests in medical offices	OHCA in areas with higher SES is associated with increased rates of B-CPR.
[24]	Retrospective cohort	3179	Non-traumatic OHCA	Increased socioeconomic status at the location of cardiac arrest is associated with increased likelihood of B-CPR
[25]	Retrospective cohort	2630	All cardiac arrests; excluded medical facility arrests, EMS-witnessed	OHCA in geographic clusters with higher SES had higher rates of B-CPR
[26]	Retrospective cohort	2028	OHCA, excluded if non-cardiac, EMS-witnessed, occurred in a medical facility	Cardiac arrests in the census tracts that rank in the highest income quintile, as compared to the lowest income quintile were much more likely to receive B-CPR
[30]	Retrospective cohort	7707	Cardiac arrests occurring in a residential dwelling, not witnessed by EMS	There is an association between B-CPR and incremental property value

SES = socioeconomic status, CPR = cardiopulmonary resuscitation, B-CPR = bystander CPR, OHCA = out-of-hospital cardiac arrest, EMS = emergency medical services.

**Table 5:** Race and Ethnicity.

Citation	Method	N	Population	Outcome
[29]	Cross-sectional ecologic study	15,109,467	Age 15 to 80, AHA, Red Cross, and Health & Safety Institute trainings	Counties with higher proportions of Black and Hispanic residents had lower CPR training rates
[32]	Retrospective cohort	18,927	Adult victims, 18 or older, non-traumatic, cardiac etiology	OHCA in predominantly Hispanic neighborhoods were less likely to receive B-CPR and had a lower likelihood of survival
[31]	Retrospective cohort	1466	OHCA for whom resuscitation was attempted by EMS	Low rates of B-CPR were associated with Black racial composition
[33]	Retrospective cohort	1068	Adult (18 and older), non-traumatic cardiac arrest	Black victims of OHCA received B-CPR less frequently than Whites
[25]	Retrospective cohort	2630	All cardiac arrests; excluded medical facility arrests, EMS-witnessed	OHCA in geographic clusters with higher percentage of White residents had higher rates of B-CPR
[34]	Retrospective cohort	1379	Non-traumatic OHCA in Arizona, excluded cases where ethnicity was not documented	Hispanic victims were less likely to receive B-CPR than non-Hispanics

CPR = cardiopulmonary resuscitation, B-CPR = bystander CPR, OHCA = out-of-hospital cardiac arrest.

**Table 6:** Language and Perceptions.

Citation	Method	N	Population	Outcome
[35]	Internet search	116	Google, Yahoo!, and Youtube searched “resucitacion cardiopulmonar”, education of CPR	16% of CPR training websites had Spanish language
[36]	Cross-sectional survey	667	Cambodians 20–64 years old, man and woman interviewed in each household	A higher level of English proficiency and greater proportion of time in the US was a strong predictor of CPR training and intention to call 9-1-1 in an emergency.
[38]	Focus group	42	Residents in high-risk census tracts (racial and SES)	Three major barriers to learning CPR were identified and included financial, informational, and motivational factors. Four major barriers were identified for performing CPR and included fear of legal consequences, emotional issues, knowledge, and situational concerns.
[37]	Focus group	64	Residents from low-income, Latino neighborhoods	Barriers to B-CPR include distrust of law enforcement, language concerns, lack of recognition of cardiac arrest, and financial issues
[39]	Focus group	46	First-generation Chinese immigrants	Barriers to B-CPR include complexity of CPR procedure, motivations to call 9-1-1, lack of confidence, and possible liability

SES = socioeconomic status, CPR = cardiopulmonary resuscitation, B-CPR = bystander CPR, EMS = emergency medical services.

## Data Availability

Not applicable.

## Supplementary Materials

The following supporting information can be downloaded at https://www.mdpi.com/article/10.3390/healthcare12040456/s1, Table S1: CPR—training and outcomes; Figure S1: PRISMA Flow Diagram.
