# Supplementary material for: Gender, Socioeconomic Status, Race, and Ethnic Disparities in Bystander Cardiopulmonary Resuscitation and Education—A Scoping Review
Source: Healthcare (Basel). 2024 Feb 10;12(4):456. doi: 10.3390/healthcare12040456 (PMC10887971; doi:10.3390/healthcare12040456)
Supplement: Supplementary file 1 [file healthcare-12-00456-s001.zip › healthcare-2662123-supplementary-TableS1.pdf]

Supplemental Table S1

Topic: CPR, training and outcomes

Searcher: SJK

Date: 10.10.19, updated 6.16.2020

Database (including vendor/platform): PubMed/NLM updated in new PubMed

Set #		Results
1CPR	"Cardiopulmonary Resuscitation"[MeSH Terms] OR "out-of-hospital cardiac arrest"[MeSH Terms] OR "Defibrillators"[Mesh] OR resuscitation[tiab] OR resuscitate[tiab] OR resuscitated[tiab] OR CPR[tiab] OR BCPR[tiab] OR AED[tiab] OR "Automated External Defibrillator"[tiab] OR "Automated External Defibrillators"[tiab] OR "out of hospital cardiac arrest"[tiab]	96112
2 Bystander	Bystander[tiab] OR bystanders[tiab] OR layperson[tiab] OR laypeople[tiab] OR laypersons[tiab]	12865
3	1 AND 2	2134
4 Education	"Health education"[mesh] OR "Certification"[Mesh] OR Methods[sh] OR education[sh] OR education[tiab] OR training[tiab] OR trained[tiab] OR train[tiab] OR educated[tiab] OR educating[tiab] OR educates[tiab] OR educate[tiab] OR certification[tiab] OR certificate[tiab] OR certified[tiab] OR certify[tiab]	4,799,560
5 Disparities	"Healthcare disparities"[mesh] OR "Sex factors"[mesh] OR "risk factors"[mesh] OR "Socioeconomic factors"[mesh] OR "Hispanic americans"[mesh] OR "African Americans"[mesh] OR disparity[tiab] OR disparities[tiab] OR socioeconomic[tiab] OR racial[tiab] OR race[tiab] OR ethnic[tiab] OR gender[tiab] OR cultural[tiab] OR "African American"[tiab] OR black[tiab] OR latino[tiab] OR latinos[tiab] OR Hispanic[tiab] OR latinx[tiab] OR Latina[tiab] OR Asian[tiab] OR income[tiab] OR wealth[tiab] OR poverty[tiab] OR barrier[tiab] OR barriers[tiab] OR education[tiab] OR educational[tiab] OR educated[tiab]	2,754,164

6	4 AND 5	873851
7	3 AND 6	437
8		

Searcher: SJK

Date: 6.17.2020

Database (including vendor/platform): Embase

Set #		Results
1CPR	'resuscitation'/exp OR 'out of hospital cardiac arrest'/exp OR 'defibrillator'/exp OR resuscitation:ti,ab OR resuscitate:ti,ab OR resuscitated:ti,ab OR CPR:ti,ab OR BCPR:ti,ab OR AED:ti,ab OR "Automated External Defibrillator":ti,ab OR "Automated External Defibrillators":ti,ab OR "out of hospital cardiac arrest":ti,ab	225244
2 Bystander	Bystander:ti,ab OR bystanders:ti,ab OR layperson:ti,ab OR laypeople:ti,ab OR laypersons:ti,ab	16992
3	1 AND 2	3963
4 Education	'health education'/exp OR 'certification'/exp OR education:lnk OR education:ti,ab OR training:ti,ab OR trained:ti,ab OR train:ti,ab OR educated:ti,ab OR educating:ti,ab OR educates:ti,ab OR educate:ti,ab OR certification:ti,ab OR certificate:ti,ab OR certified:ti,ab OR certify:ti,ab	1517157
5 Disparities	'health care disparity'/exp OR 'sex factor'/exp OR 'risk factor'/exp OR 'socioeconomics'/exp OR 'Hispanic'/exp OR 'Black person'/exp OR disparity:ti,ab OR disparities:ti,ab OR socioeconomic:ti,ab OR racial:ti,ab OR race:ti,ab OR ethnic:ti,ab OR gender:ti,ab OR cultural:ti,ab OR "African American":ti,ab OR black:ti,ab OR latino:ti,ab OR latinos:ti,ab OR Hispanic:ti,ab OR latinx:ti,ab OR Latina:ti,ab OR Asian:ti,ab OR income:ti,ab OR wealth:ti,ab OR poverty:ti,ab OR barrier:ti,ab OR barriers:ti,ab OR education:ti,ab OR educational:ti,ab OR educated:ti,ab	3304005

6	4 AND 5	777559
7	3 AND 6	632
8	AND [humans]/lim	598
