# Supplementary figures and images for: Gender, Socioeconomic Status, Race, and Ethnic Disparities in Bystander Cardiopulmonary Resuscitation and Education—A Scoping Review
Source: Healthcare (Basel). 2024 Feb 10;12(4):456. doi: 10.3390/healthcare12040456 (PMC10887971; doi:10.3390/healthcare12040456)

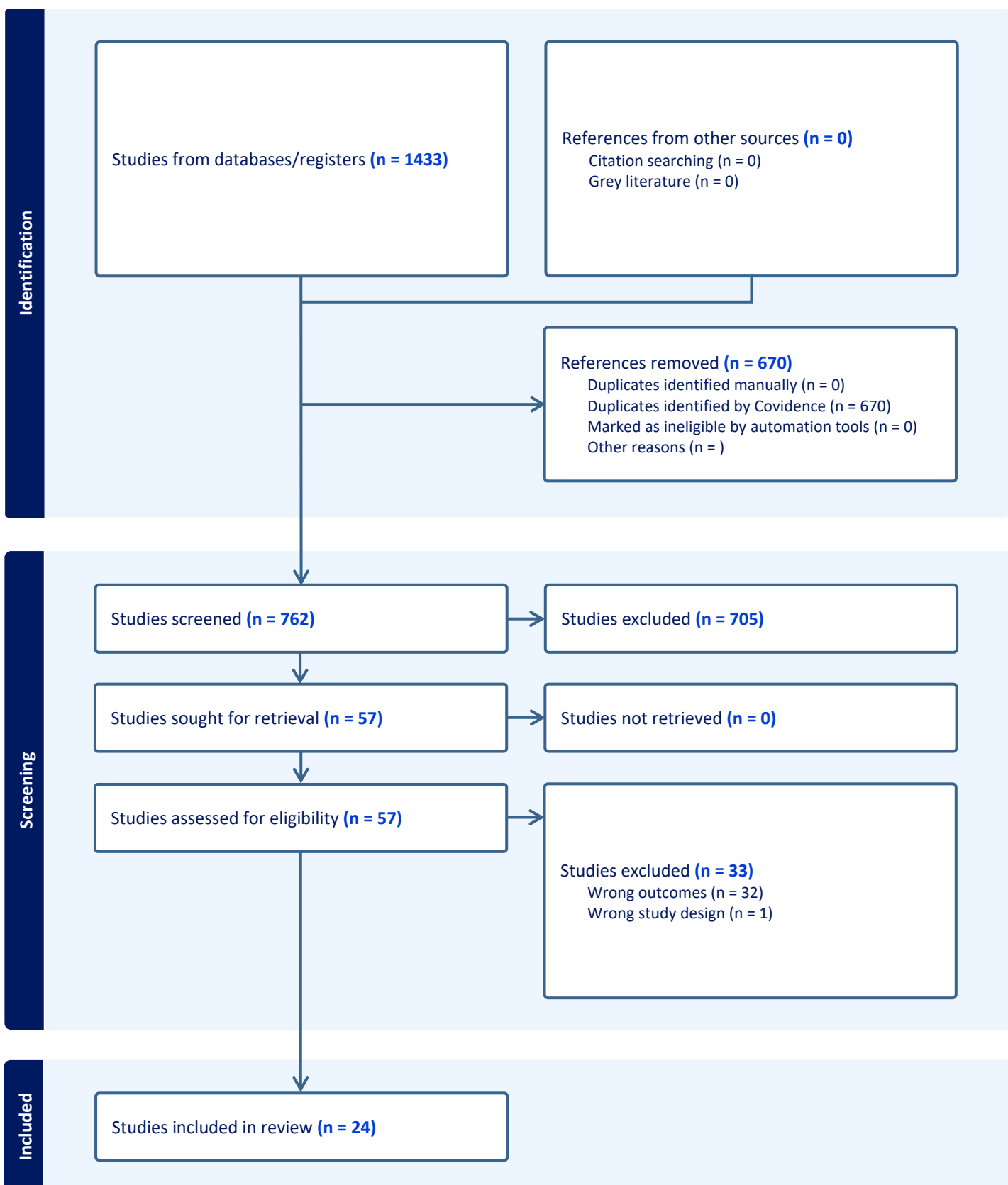

Supplement: Supplementary file 1 [file healthcare-12-00456-s001.zip › healthcare-2662123-supplementary-FigureS1.pdf]
